# Inhibition of AMPK activity by TRIM11 facilitates cell survival of hepatocellular carcinoma under metabolic stress

**DOI:** 10.1002/ctm2.617

**Published:** 2021-12-17

**Authors:** Yanfeng Liu, Yingying Xu, Fan Wang, Yu Tong, Hongchang Li, Xiaochun Wan, Xiaolu Yang, Liang Chen

**Affiliations:** ^1^ State Key Laboratory of Oncogenes and Related Genes, Renji‐Med‐X Clinical Stem Cell Research Center, Department of Liver Surgery, Ren Ji Hospital, School of Medicine Shanghai Jiao Tong University Shanghai China; ^2^ Shenzhen Laboratory of Tumor Cell Biology, Center for Protein and Cell‐based Drugs, Institute of Biomedicine and Biotechnology Shenzhen Institute of Advanced Technology, Chinese Academy of Sciences Shenzhen China; ^3^ Department of Cancer Biology and Abramson Family Cancer Research Institute, Perelman School of Medicine University of Pennsylvania Philadelphia Pennsylvania USA

To the Editor:

AMP‐responsive protein kinase (AMPK) is a master nutrient and energy sensor, keeping the cellular energy homeostasis during metabolic stress.^1^, ^2^ Loss of AMPK or deregulation of its activity has been detected in multiple human cancers including hepatocellular carcinoma (HCC).^3^, ^4^ However, the underlying molecular mechanism of dysregulation of AMPK activity is still largely unclear. We previously demonstrated that an E3 ubiquitin‐ligase, TRIM11, is a key player during various stress conditions and tumourigenesis.^5^, ^6^ In this report, we clarified TRIM11 as a new mechanism for negatively regulating AMPK activity during glucose starvation, and suggest that TRIM11‐AMPK axis is crucial for HCC survival and progression.

We determined that TRIM11 is liked with metabolic reprogramming and its expression was induced upon glucose starvation (Figure 1A and Figure S1A–C). As glucose deprivation would be detrimental for tumour cell survival, we examined the role of TRIM11 in this process and found it positively regulated tumour cells viability upon glucose deprivation (Figure 1B,C and Figure S1D–F), and similar results could be found in the role of TRIM11 in vivo (Figure 1D–F). Gene set enrichment analysis (GSEA) revealed that several metabolic pathways gene set were significantly enriched in the TRIM11_low expression HCC patients (Figure 1G and Figure S1G and Table S1). Then, we indeed found that TRIM11 could regulate glucose metabolism in the HCC cells and patient tissues (Figure 1H–J and Figure S1H). Together, these results suggest that the upregulation of TRIM11 serves as a novel protecting mechanism to avoid HCC cells death upon metabolic stress.

**FIGURE 1:**
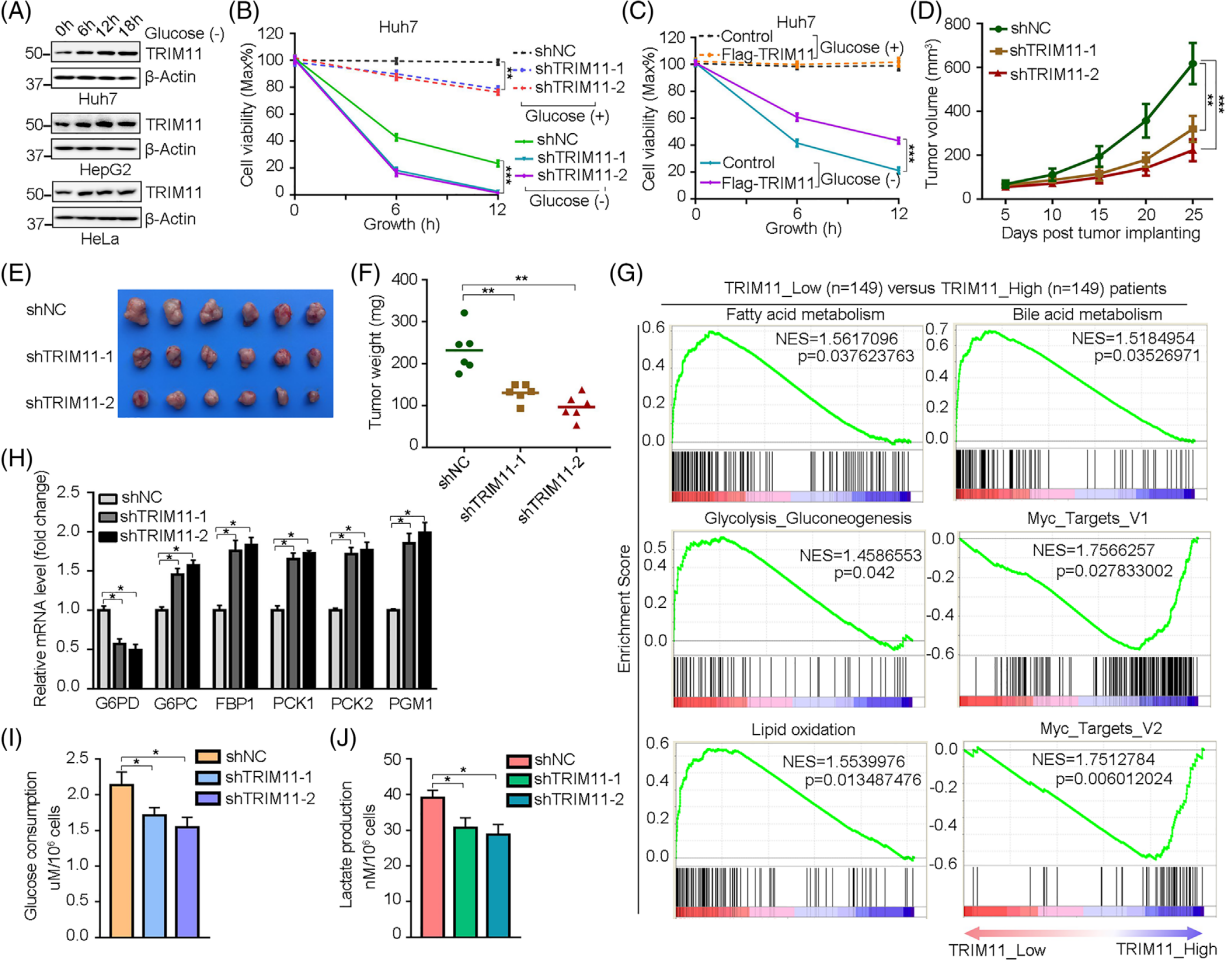
TRIM11 functions as a feedback mechanism that responds to metabolic stress in the HCC progression. (A) Western blot analysis of TRIM11 in Huh7, HepG2 and Hela cells treated with glucose starvation (indicated time). (B and C) Cell viability of Huh7 cells stably expressing control or TRIM11 shRNA (B), and control or F‐TRIM11 (C), treated with or without glucose starvation for 6 or 12 h. (D–F) Huh7 cells stably expressing the indicated shRNAs (shNC, shTRIM11‐1 and shTRIM11‐2) were subcutaneously injected in nude mice. Shown are average tumour volumes over time (*n* = 6) (D), representative image (E) and weights (F) of tumours at day 25. (G) Fatty acid metabolism, bile acid metabolism, glycolysis/gluconeogenesis and lipid oxidation were significantly enriched and MYC targets were remarkably compromised in the TRIM11_low expression group. (H) Relative glucose metabolic genes (G6PD, G6PC, FBP1, PCK1, PCK2 and PGM1) mRNA fold change in the Huh7 cells with control (shNC) or stable knockdown of TRIM11. (I and J) Glucose consumption (I) and lactate production (J) of Huh7 cells infected with shRNA targeting TRIM11 were determined. For (B–D), (F) and (H–J), data represent the mean ± SEM. Statistical significance was assessed using two‐tailed Student's *t*‐tests. *p < .05, **p < .01, ***p < .001; n.s., not significant

Next, we analysed the protein interaction network of TRIM11 and demonstrated that TRIM11 mainly interacts with AMPKβ2 and also shows a weak interaction with AMPKα and AMPKγ2 subunits (Figure 2A and Figure S2A–D). AMPK consists of catalytic α, regulatory β and γ subunits, and all of these subunits are closely linked to the activating AMPK,^7^, ^8^ implying TRIM11 may regulate AMPK activity by selective modulation of AMPK regulatory subunits. As expected, TRIM11 significantly enhanced ubiquitination level of AMPKβ2 and accelerated its protein degradation (Figure 2B,C and Figure S2E), and negatively controlling AMPK signaling activity in the HCC cells and tissues (Figure 2D,E and Figure S2F–L). To map the binding domain between TRIM11 and AMPKβ2, we constructed their corresponding deletion mutants (Figure S2M,N), and determined that the RING domain of TRIM11 is required for the interaction with AMPKβ2 (Figure 2F). As we previously reported,^5^, ^6^ a TRIM11 mutant (TRIM11‐2CA) that lost its ubiquitination activity was used (Figure S2M). TRIM11‐2CA displayed a reduced interaction with AMPKβ2, decreased its ubiquitination, increased stability of AMPKβ2, and also impaired its function in controlling AMPK activity and tumour cell viability (Figure 2G–K). Meantime, we found that the β‐CTD of AMPKβ2 was crucial for its interaction with TRIM11 and that the AMPKβ2‐K260R mutant could suppress the ubiquitination level of AMPKβ2 and enhance its stability (Figure 2L,M and Figure S2O), suggesting that TRIM11 directly targets K260 of AMPKβ2 to mediate its degradation. Collectively, these data demonstrate that TRIM11 destabilizes AMPKβ2 through directly promoting its protein degradation, which is required for its effects on AMPK activity and HCC cell survival.

**FIGURE 2:**
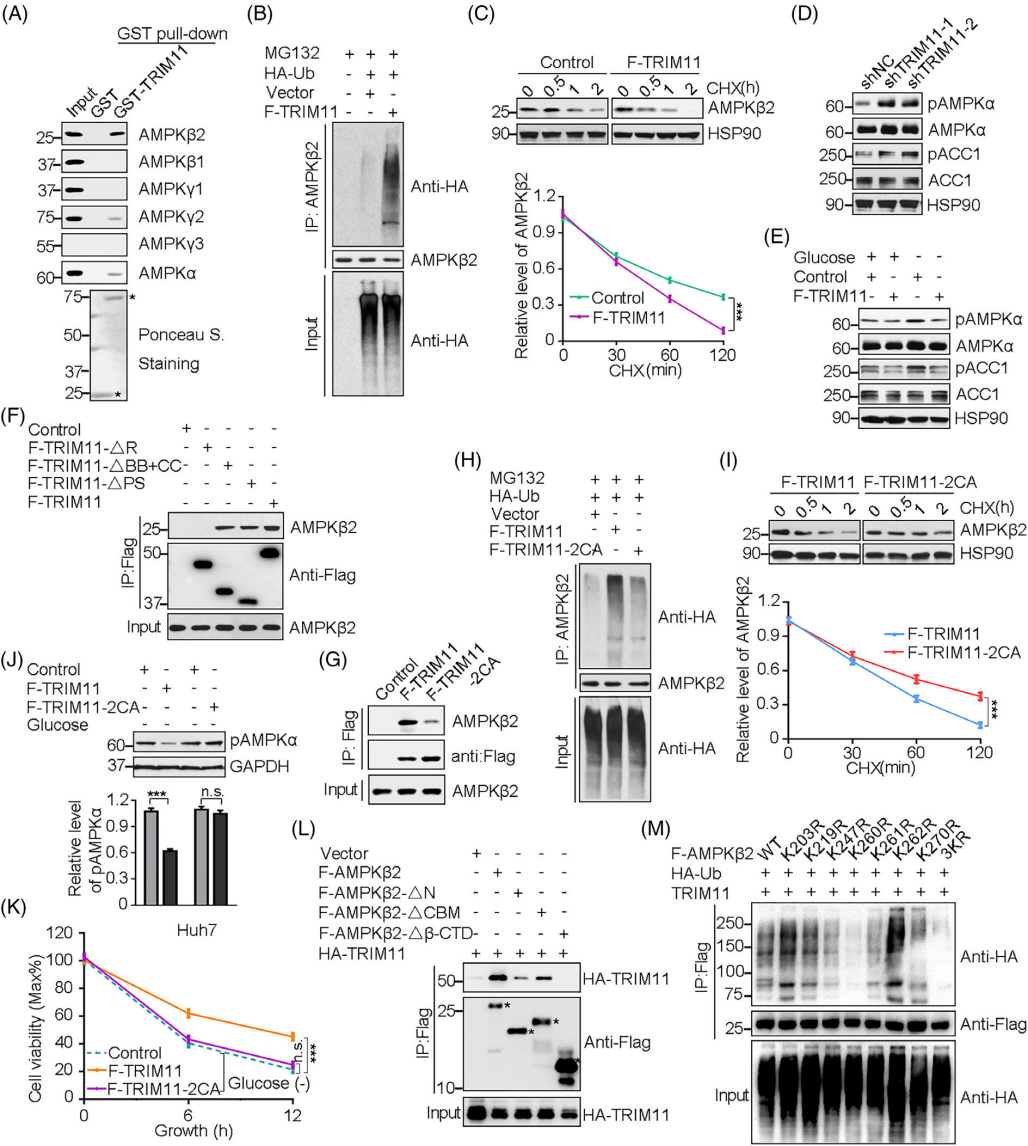
TRIM11 binds to and ubiquitinates AMPKβ2 subunit, negatively regulates AMPK activity and details of TRIM11‐AMPK binding. (A) Interaction of TRIM11 with AMPK subunits, AMPKβ2, AMPKβ1, AMPKγ1, AMPKγ2, AMPKγ3 and AMPKα. AMPKα antibody detects both AMPKα1 and AMPKα2 subunits. Beads‐conjugated GST and GST‐TRIM11 (1 μg each) were incubated with Huh7 cell lysates treated with MG132 (2 μM) and were analysed by Western blot with indicated antibody or Ponceau S staining. Asterisk indicates GST or GST‐TRIM11. (B) Western blot analysis of AMPKβ2 IP in control and Flag‐TRIM11 (F‐TRIM11)‐expressing Huh7 cells, transfected with HA‐ubiquitin (HA‐Ub) and treated with MG132 (2 μM) for 8 h. Cell lysates were first denatured in SDS‐containing buffer and diluted in nondenaturing buffer before being incubated with anti‐AMPKβ2 antibody. (C) Analysis of AMPKβ2 half‐life in control and F‐TRIM11‐expressing Huh7 cells. Cells were treated with cycloheximide (CHX, 100 mg/ml) at the indicated times to inhibit new protein synthesis and analysed by Western blot. Representative Western blot and relative level of AMPKβ2 is shown. (D) Representative Western blot analysis of the levels of phospho‐AMPKα/AMPKα and phospho‐ACC1/ACC1 in Huh7 cells stably knocking down control (shNC) or TRIM11. (E) Representative Western blot analysis of the levels of phospho‐AMPKα/AMPKα and phospho‐ACC1/ACC1 in Huh7 cells stably overexpressing control or F‐TRIM11, treated with or without glucose starvation. (F) Interaction of Flag‐TRIM11 proteins with endogenous AMPKβ2 in Huh7 cells was analysed by co‐IP assay. (G) Interaction of Flag‐TRIM11 and Flag‐TRIM11‐2CA with endogenous AMPKβ2 in Huh7 cells was analysed as indicated. (H) Western blot analysis of AMPKβ2 IP in control, F‐TRIM11‐ or F‐TRIM11‐2CA‐expressing Huh7 cells, transfected with HA‐Ub and treated with MG132 (2 μM) for 8 h. Cell lysates were first denatured in SDS‐containing buffer and diluted in nondenaturing buffer before being incubated with anti‐AMPKβ2 antibody. (I) Analysis of AMPKβ2 half‐life in F‐TRIM11‐ or TRIM11‐2CA‐expressing Huh7 cells. Cells were treated with cycloheximide (CHX, 100 mg/ml) at the indicated times to inhibit new protein synthesis and analysed by Western blot. Representative Western blot (top) and relative AMPKβ2/HSP90 ratios (low) are shown. (J) Western blot analysis of the levels of phospho‐AMPKα (pAMPK) in U2OS cells stably overexpressing control, F‐TRIM11 or F‐TRIM11‐2CA, treated with glucose starvation. Representative Western blot (top) and relative pAMPK (low) are shown. (K) Cell viability of Huh7 cells stably expressing control, F‐TRIM11 or F‐TRIM11‐2CA, treated with glucose starvation for 6 or 12 h. (L and M) HEK293T cells were transfected with the indicated plasmids. K203R, K219R, K247R, K260R, K261R, K262R, K270R and 3KR indicate eight AMPKβ2 mutants with Lys203, Lys219, Lys247, Lys260, Lys261, Lys262, Lys270 and Lys260‐262 changed into Arg. Immunoblot analysis of the FLAG‐IP and cell lysates (L), and the ubiquitination of wide‐type and AMPKβ2 mutants with the indicated antibodies (M). For IPs/pull‐down, 10% of cell lysate as input was used. For (C), (I) and (K), data represent the mean ± SEM. Statistical significance was assessed using two‐tailed Student's *t*‐tests. ***p < .001, n.s., not significant

AMPK can be activated upon glucose deprivation, which results in starvation‐induced autophagy, this trigging autophagic cell death,^7^, ^9^ suggesting that TRIM11 may act as an upstream regulator of AMPK/autophagy pathway. We analysed that autophagy activation was inversely linked with TRIM11 level in HCC (Figure S3A). Then, we observed the formation of autophagosomes and evaluated the localisation of LC3B, a marker protein for autophagosomes,^10^ as well as the expression level of the autophagy markers (LC3‐II and p62), confirming that TRIM11 negatively regulates the induction of autophagy during metabolic stress (Figure 3A–F and Figure S3B,C). While TRIM11‐2CA was less effective in regulating the autophagy compared with TRIM11‐WT (Figure S3D). In addition, autophagic flux inhibitor chloroquine (CQ) could abrogate the effective knockdown of TRIM11‐mediated activation of autophagy (Figure S3E). Of note, TRIM11‐mediated autophagy regulation and tumour cell survival were diminished in AMPK‐knockdown cells or treated with AMPK inhibitor compound C (Figure S3F–N), revealing that TRIM11‐mediated negative regulation of autophagy depends on AMPK. Similarly, we confirmed this conclusion in MEFs (Figure 3G–J and Figure S3O,P). Together, these data demonstrate that TRIM11 negatively regulates autophagy depending on its controlling AMPK activity.

**FIGURE 3:**
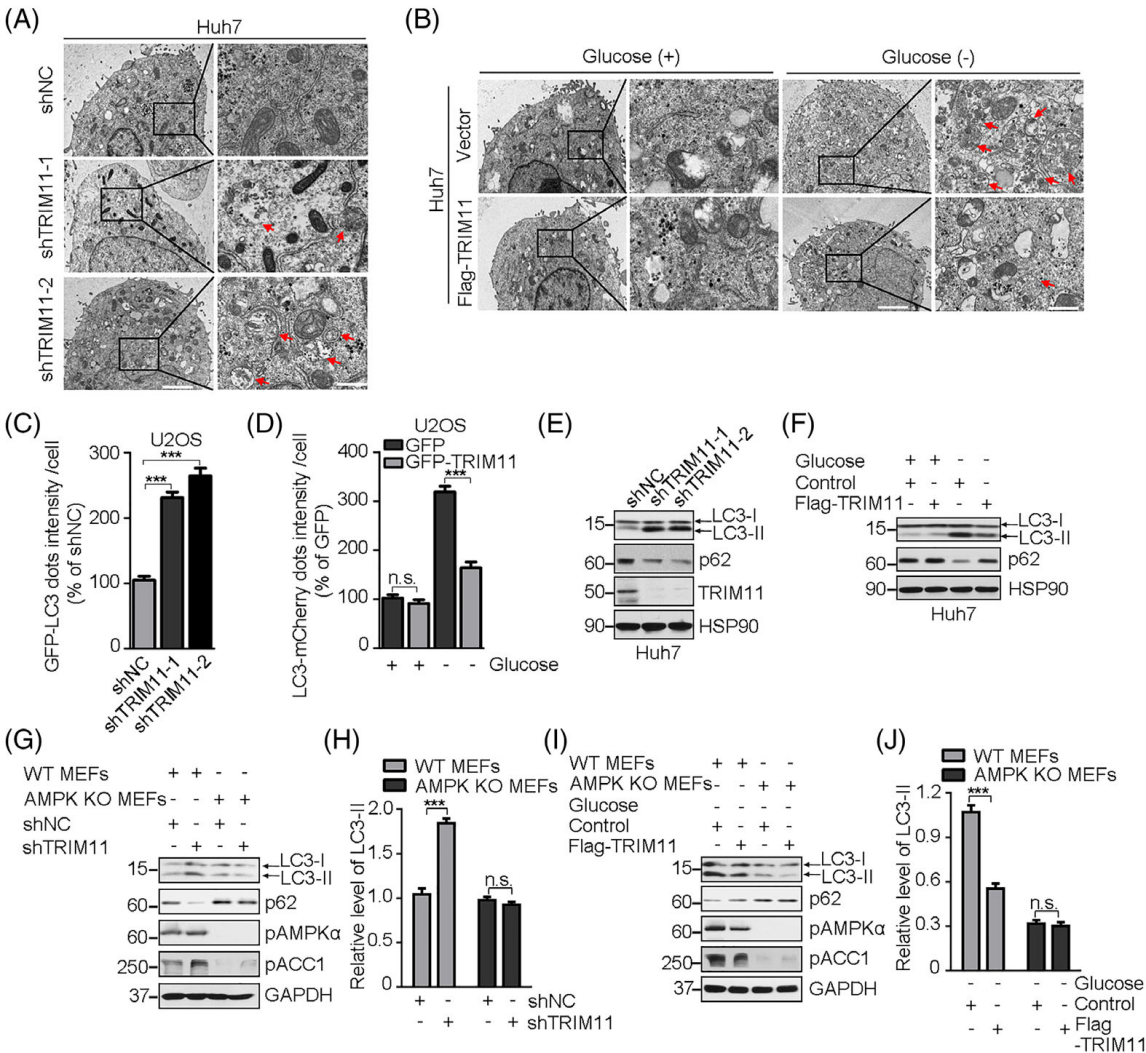
TRIM11 inhibits glucose deprivation‐induced autophagy stimulation depending on its controlling AMPK activity. (A and B) Electron microscope images of autophagosomes in Huh7 cells (A) stably transfected with a control shRNA (shNC) or one of the two independent TRIM11 shRNAs, and cells (B) stably expressing vector or F‐TRIM11 with treatment of mock or glucose starvation. The black square region was correspondingly magnified on the right. The red arrow indicates the formation of autophagosomes. Scale bar: left, 2 μm; right, 0.5 μm. (C and D) Quantification of GFP‐LC3 dots in U2OS cells stably transfected with a control shRNA (shNC) or one of the two independent TRIM11 shRNAs (C), and LC3‐mCherry dots in U2OS cells stably expressing vector or F‐TRIM11 with treatment of mock or glucose starvation (D); 250 (C) or 200 (D) cells from each indicated sample were quantified and the relative intensity was shown. (E and F) Western blot analysis of p62 and LC3 levels in Huh7 cells stably knocking down control or TRIM11 (E), and cells stably expressing vector or F‐TRIM11 with treatment of mock or glucose starvation (F). LC3‐I and LC3‐II are indicated by arrows. (G and H) Representative Western blot (G) and quantification (H) analysis of the levels of LC3 in control or AMPK knockout (KO) MEF cells with stably knocking down control or TRIM11. LC3‐I and LC3‐II are indicated by arrows. (I and J) Representative Western blot (I) and quantification (J) analysis of the levels of LC3 in control or AMPK KO MEF cells with stably expressing control or TRIM11, incubated with glucose starvation medium. LC3‐I and LC3‐II are indicated by arrows. For (C), (D), (H) and (J), data represent the mean ± SEM (*n* = 3 unless otherwise indicated). Statistical significance was assessed using two‐tailed Student's *t*‐tests. ***p < .001; n.s., not significant

Next, we explored whether AMPK activity is required for TRIM11‐mediated protection of HCC cell survival. As expected, TRIM11 indeed significantly enhanced HCC cell viability upon the AMPK activator AICAR treatment, but TRIM11‐mediated HCC cell survival was abrogated in the AMPK‐knockdown cells (Figure S4A,B). Consistently, this effect was also confirmed in MEFs (Figure 4A). And TRIM11 also obviously abrogate metformin‐mediated HCC therapeutic effects in vivo (Figure 4B,C and Figure S4C–E),suggesting that inhibiting TRIM11‐AMPK axis helps effective treatment of HCC. For its clinical significance in HCC patients, we found that the expression level of TRIM11 negatively correlated with AMPKβ2 and pAMPK (Figure 4D and Figure S4F,G). In addition, we found an increased TRIM11 staining intensity in HCC tissues relative to adjacent counterparts (Figure S5A,B), and the transcription levels of TRIM11 were also significantly upregulated in many cancerous tissues (Figure S5C–J). Of note, survival analysis showed that TRIM11^High^ predicts worse overall survival than those with TRIM11^Low^ (Figure 4E,F). Similar results were found in the TCGA cohort (Figure 4G and Figure S5K–N). Consistently, TCGA pan‐cancer cohort also revealed TRIM11^High^ in the tumour patients displayed shorten overall survival (OS) and disease/progression‐free survival time (DFS/PFS) compared with TRIM11^Low^ tumour patients (Figure 4H and Figure S5O,P). Thus, these results demonstrate that upregulation of TRIM11 promotes tumour progression and can be served as a crucial indicator for poor prognosis in the pan‐cancer cohort.

**FIGURE 4:**
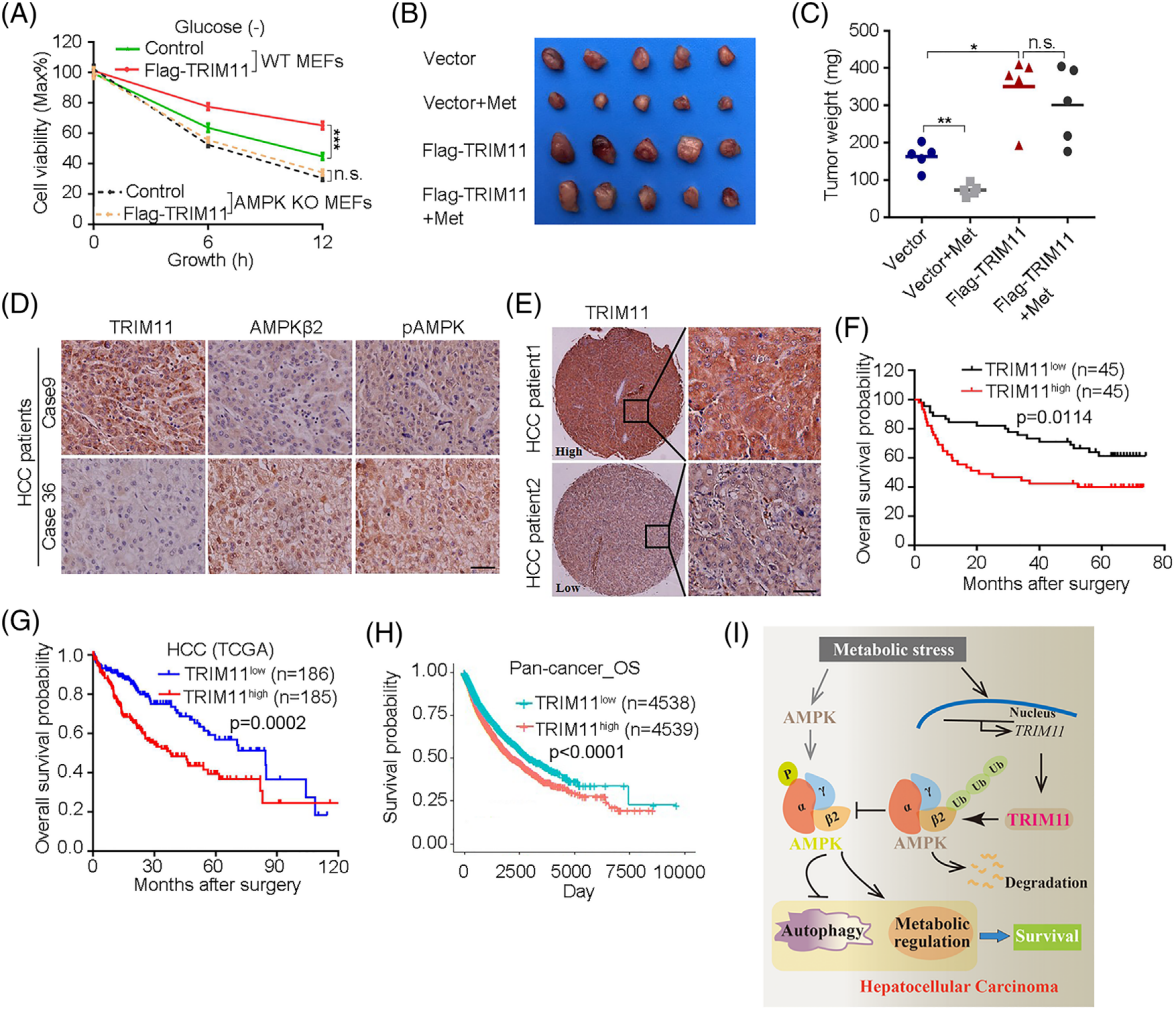
TRIM11 facilitates tumour cell growth during glucose deprivation by regulating AMPK activity and upregulation of TRIM11 in HCC and pan‐cancer linked to poor prognosis. (A) Cell viability of control or AMPK knockout (KO) MEF cells with stably expressing control or TRIM11, treated with or without glucose starvation for 6 or 12 h. (B and C) Huh7 cells stably expressing vector or F‐TRIM11 were subcutaneously injected in nude mice, which were fed with control (tap water) or metformin water (Met, 500 mg/L), respectively. Shown are representative image (B) and weights (C) of tumours at day 25. (D) Representative images of IHC staining of TRIM11, Ki67, AMPKβ2 and pAMPK in HCC tissues of mice inoculated with vector or F‐TRIM11, treated with control and metformin, respectively. Scale bar: 50 μm. (E and F) Representative images of IHC (E) and Kaplan–Meier analysis of overall survival (OS) probability (F) of TRIM11 levels in HCC patients. The statistical significance was assessed using log‐rank test according to HCC patients with low or high expression of TRIM11 (*n* = 45). Scale bar: 50 μm. (G) The OS probability was compared between TRIM11 high (*n* = 185) and low expression (*n* = 186) in HCC patients from TCGA cohort. (H) The OS was compared between TRIM11 high (*n* = 4539) and low expression (*n* = 4538) in the 33 of the most prevalent forms of cancer patients from TCGA pan‐cancer cohort. (I) Schematic diagram shows the work model of TRIM11‐AMPK axis in HCC progression. TRIM11 is transcriptionally activated upon metabolic stress (e.g., glucose deprivation), which inhibits AMPK activity through directly ubiquitinates and degrades AMPKβ2 subunit, leading to autophagy suppression and metabolic regulation, and finally helps HCC cell survival and growth during this stress. For (A) and (C), data represent the mean ± SEM (*n* = 3 unless otherwise indicated). Statistical significance was assessed using two‐tailed Student's *t*‐tests. *p < .05, **p < .01, ***p < .001; n.s., not significant; for (F–H), the statistical significance was assessed using two‐sided log‐rank test; log‐rank *p*‐values are shown

Together, our data demonstrated that TRIM11 was found to be significantly induced to mediate cellular metabolic reprogramming and inhibited the stimulation of autophagy via directly targeting AMPK signaling pathway to promote HCC cell survival (Figure 4I). Our study highlights a crucial promoter implicated in metabolic stress, the TRIM11‐AMPK axis, which will provide the theoretical basis and intervention targets for developing more effective means of clinical treatment of HCC.

## CONFLICT OF INTEREST

The authors declare that there is no conflict of interest.

## Supporting information

Supporting Information

Table S1
