# Supplementary material for: Inhibition of AMPK activity by TRIM11 facilitates cell survival of hepatocellular carcinoma under metabolic stress
Source: Clin Transl Med. 2021 Dec 17;11(12):e617. doi: 10.1002/ctm2.617 (PMC8679837; doi:10.1002/ctm2.617)
Supplement: Supplementary file 1 — Supporting Information [file CTM2-11-e617-s001.docx]

**
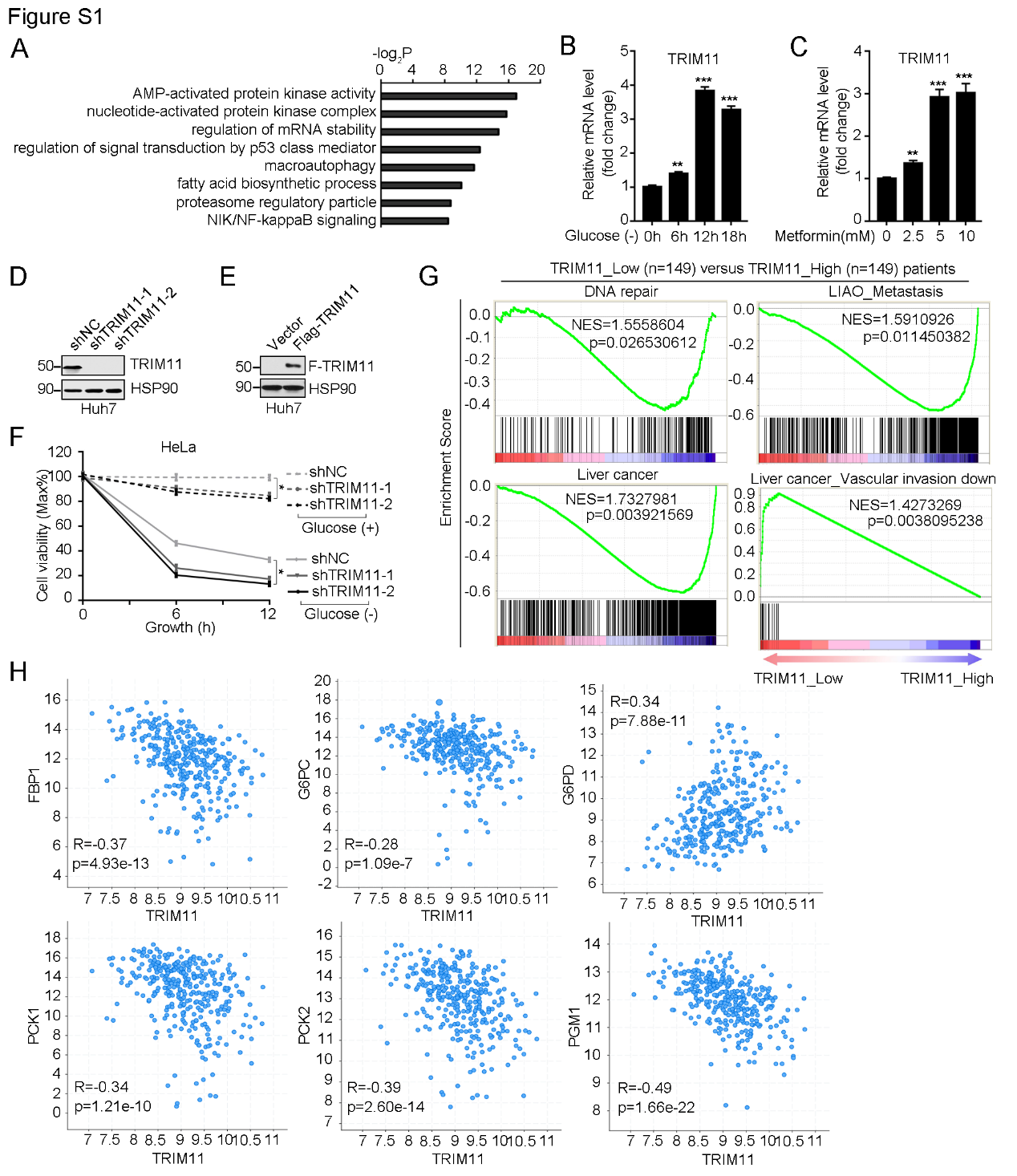
**

Supplementary Figure S1. The expression of TRIM11 is induced by metabolic stress, associated with liver cancer progression and metabolic reprogramming processes.

(A) Gene ontology (GO) of TRIM11 analyzed by BioPlex 2.0 (<http://bioplex.hms.harvard.edu/bioplexDisplay/index.php>). The most significant network GO enrichments were shown. (B, C) Relative mRNA fold change of TRIM11 in Huh7 cells treated with glucose starvation (indicated time) (B) or metformin-contained (indicated dose) normal medium (C). (D) Western blot analysis of control and Flag-TRIM11-expressing (F-TRIM11) Huh7 cells. (E) Western blot analysis of the Huh7 cells stably knocking down control (shNC) or TRIM11. (F) Cell viability of Hela cells stably expressing control or TRIM11 shRNA treated with or without glucose starvation for 6h or 12h. (G) GSEA analysis showed that DNA repair, LIAO_Metastasis and Liver cancer signatures were significantly enriched and liver cancer vascular invasion down gene set was remarkably compromised in the TRIM11_high expression HCC patients (n=149) compared with the TRIM11_low expression group (n=149). The HCC patient’s tissue date comes from the TCGA_LIHC database. (H) TCGA-LIHC database analyses the expression correlation between TRIM11 and glucose metabolic genes (G6PD, G6PC, FBP1, PCK1, PCK2 and PGM1) by Pearson’s correlation. For B, C and F, data represent the mean ± SEM. Statistical significance was assessed using two-tailed Student’s t-tests. *, P< 0.05, **, *P* < 0.01, ***, P <0.001.


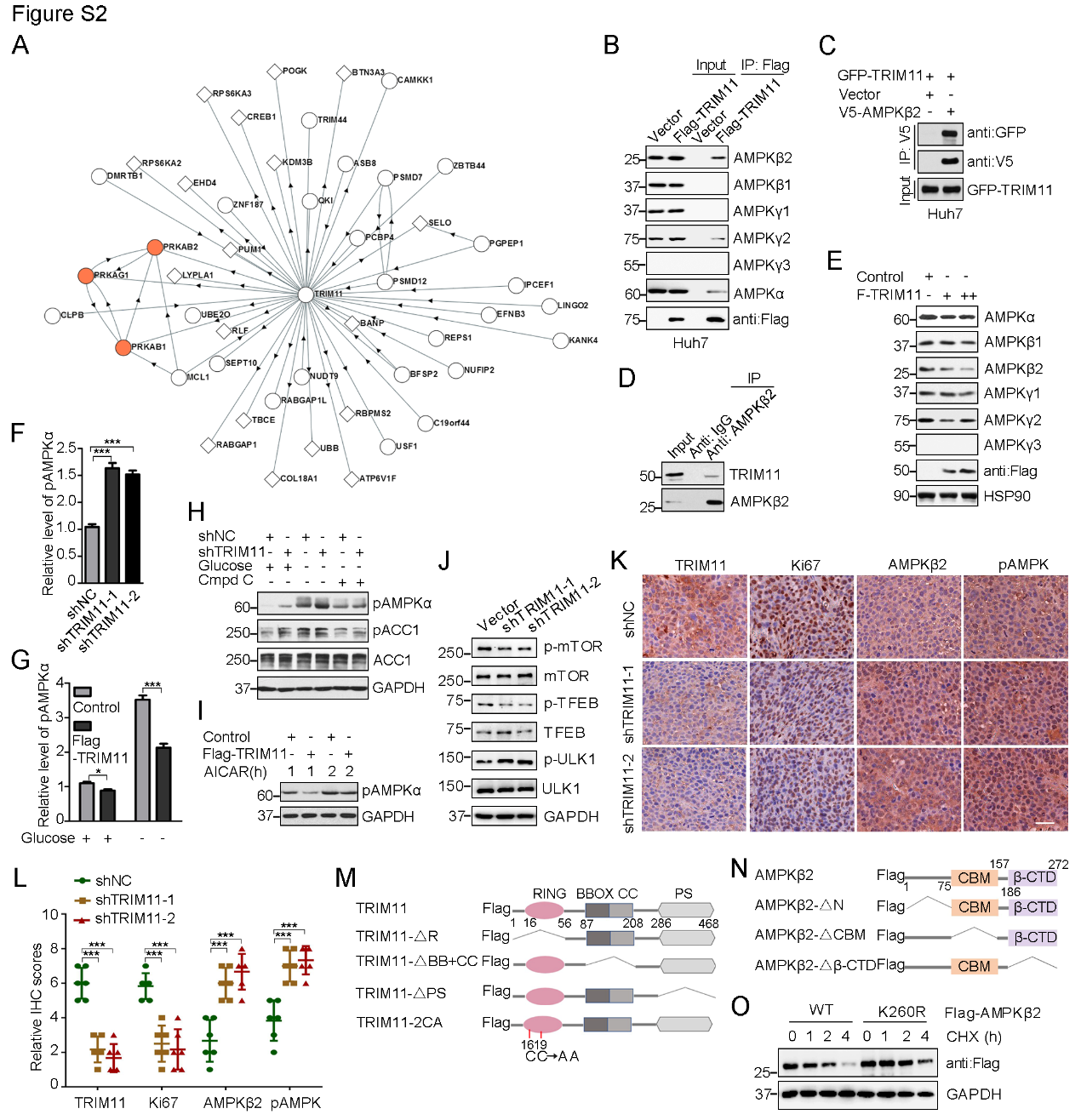


Supplementary Figure S2. TRIM11 specially associates with AMPKβ2 and negatively regulates AMPK activity. (A) Interaction network of TRIM11 analyzed by BioPlex 2.0 (<http://bioplex.hms.harvard.edu/bioplexDisplay/index.php>). The potential associated candidates of AMPK subunits were labelled in orange. (B) Interaction of F-TRIM11 proteins with the indicated AMPK subunits in Huh7 cells treated with MG132 (2 μM) was analyzed by co-IP assay. (C) Interaction of GFP-TRIM11 with V5-AMPKβ2 in Huh7 cells was analyzed by co-IP assay. (D) Interaction of endogenous TRIM11 with AMPKβ2 in Huh7 cells was analyzed by co-immunoprecipitation (IP) assays. (E) Western blot analysis the level of AMPK subunits, AMPKα, AMPKβ1, AMPKβ2, AMPKγ1, AMPKγ2 and AMPKγ3 in Huh7 cells transfected with control or F-TRIM11 (Flag-TRIM11) (+, 0.75μg; ++, 1.5 μg). (F) Quantification analysis of the levels of phospho-AMPKα/AMPKα and phospho-ACC1/ACC1 in Huh7 cells stably knocking down control (shNC) or TRIM11. (G) Quantification analysis of the levels of phospho-AMPKα/AMPKα and phospho-ACC1/ACC1 in Huh7 cells stably overexpressing control or F-TRIM11, treated with or without glucose starvation. (H) Western blot analysis of the levels of phospho-AMPKα and phospho-ACC1/ACC1 in Huh7 cells stably knocking down control (shNC) or TRIM11, treated for 8h with or without compound C (Cmpd C, 10 μM) and incubated with or without glucose deprivation medium. (l) Western blot analysis of the levels of phospho-AMPKα in Huh7 cells stably overexpressing control or F-TRIM11, treated for 1 or 2h with or without AICAR (2 mM). (J) Western blot analysis of the levels of phospho-mTOR/ mTOR, phospho-TFEB/TFEB and phospho-ULK1/ULK1 in the Huh7 cells stably knocking down control (shNC) or TRIM11 as indicated. (K, L) Representative images (K) of IHC staining and the relative IHC scores (n=6) (L) of TRIM11, Ki67, AMPKβ2 and pAMPK in HCC tissues of mice inoculated with Huh7-shNC or Huh7-shTRIM11-1&-2. Scale bar, 50 μm. (M) Schematic diagram of TRIM11 and its domain deletion mutants/point mutation. For TRIM11-2CA, the conserved Cys16 and Cys19 were mutated to Ala, which blocked the ubiquitination activity of TRIM11. Flag epitope was tagged with each construct. (N) The carbohydrate-binding module (CBM) domain and β-subunit C-terminal domain (β-CTD) of AMPKβ2 were indicated. (O) HEK293T cells transfected with Flag-AMPKβ2 wide-type or AMPKβ2-K260R mutant were subject to cycloheximide (CHX) treatment for indicated time course and subjected to immune-blot analysis. For F, G and L, data represent the mean ± SEM. Statistical significance was assessed using two-tailed Student’s t-tests. *, P< 0.05, ***, P <0.001.


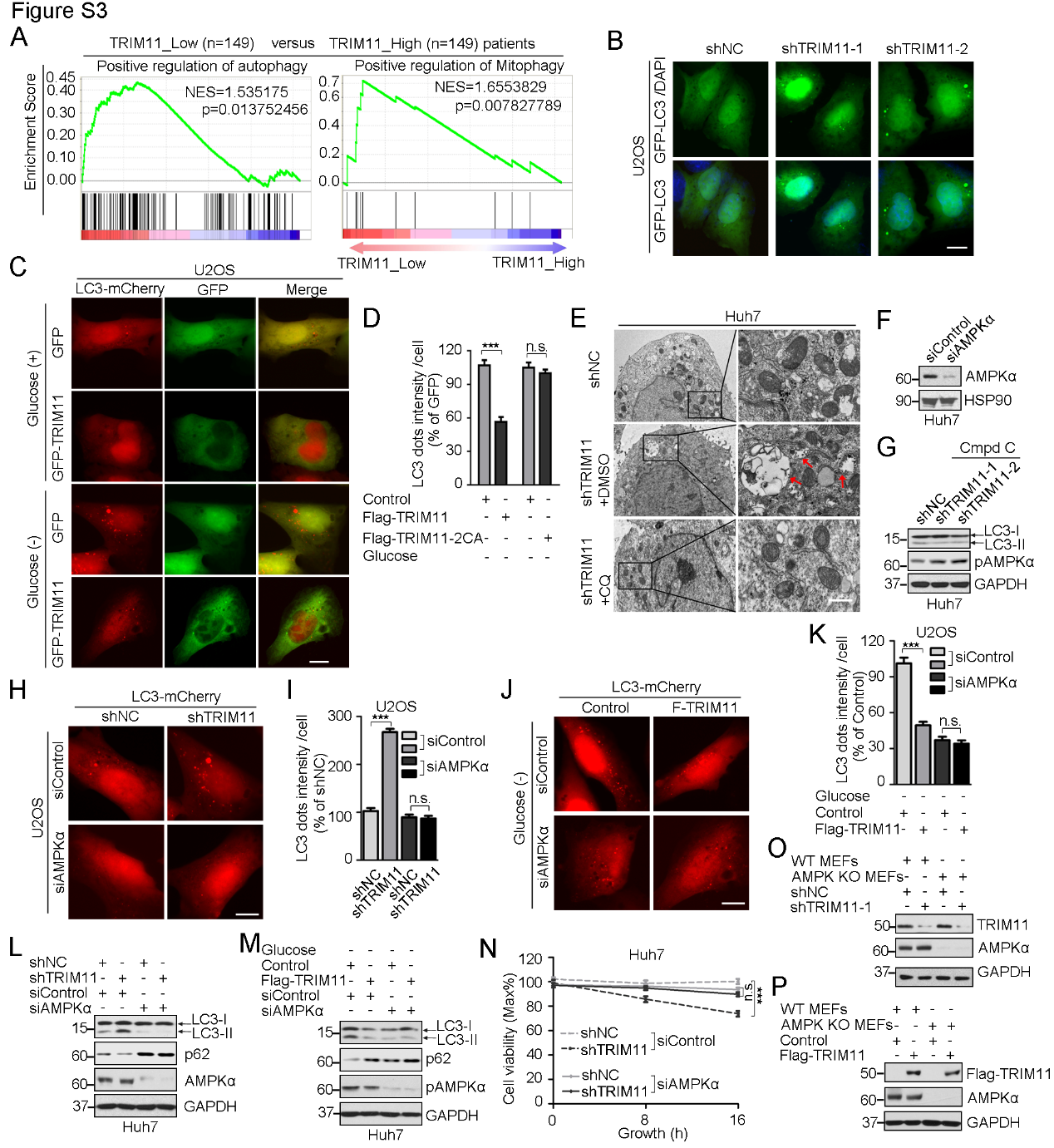


Supplementary Figure S3. TRIM11 negatively regulates autophagy and cell survival depending on AMPK. (A) Autophagy and mitophagy regulation were significantly enriched in the TRIM11_low expression HCC patients (n=149) compared with the TRIM11_high expression group (n=149). (B, C) Representative images of GFP-LC3 dots in U2OS cells stably transfected with a control shRNA (shNC) or one of the two independent TRIM11 shRNAs (B), and representative images of LC3-mCherry dots in U2OS cells stably expressing GFP or GFP-TRIM11 with treatment of mock or glucose starvation (C). Scale bar, 10 μm. (D) Quantification of GFP-LC3 dots in U2OS cells stably expressing control, F-TRIM11 or F-TRIM11-2CA, treated with glucose starvation. 200 cells from each indicated sample were quantified and the intensity of GFP-LC3 dots relative to control expressing U2OS cells. (E) Electron microscope images of autophagosomes in Huh7 cells stably transfected with a control shRNA (shNC) or the TRIM11 shRNA with treatment of DSMO or the autophagic flux inhibitor chloroquine (CQ). The black square region was correspondingly magnified on the right. The red arrow indicates the formation of autophagosomes. (F) Western blot analysis of the knocks down efficiency of AMPKα in Huh7 cells. (G) Western blot analysis of LC3 level in Huh7 cells stably knocking down control or TRIM11, treated for 8h with compound C (Cmpd C, 10 μM). LC3-I and LC3-II are indicated by arrows. (H, I) Representative images (H) and quantification (I) of LC3-mCherry dots in U2OS cells stably expressing control or TRIM11 shRNA simultaneously with knockdown of control or AMPK. 200 cells from each indicated sample were quantified and the intensity of LC3-mCherry dots relative to control expressing U2OS cells. (J, K) Representative images (J) and quantification (K) of LC3-mCherry dots in U2OS cells stably expressing control or F-TRIM11 simultaneously with knockdown of control or AMPK. 200 cells from each indicated sample were quantified and the intensity of LC3-mCherry dots relative to control expressing U2OS cells incubated with glucose deprivation medium. (L, M) Western blot analysis of LC3 and p62 level in Huh7 cells transfected with siRNA of control or AMPK simultaneously with control or TRIM11 knocking down (L) or force expression (M), incubated with complete or glucose starvation medium. LC3-I and LC3-II are indicated by arrows. (N) Cell viability of Huh7 cells stably expressing control or TRIM11 shRNA simultaneously transfected with control or AMPK siRNA, incubated with complete medium for 8h or 16h. (O, P) Western blot analysis of MEF wild type (WT) and AMPK knockout (KO) cells stably expressing control or TRIM11 shRNA (O), and control or F-TRIM11 (P). Data represent the mean ± SEM (n = 3). Statistical significance was assessed using two-tailed Student’s t-tests. ***, P <0.001, n.s., not significant. For D, I, K and N, data represent the mean ± SEM (n = 3). Statistical significance was assessed using two-tailed Student’s t-tests. ***, P <0.001, n.s., not significant.


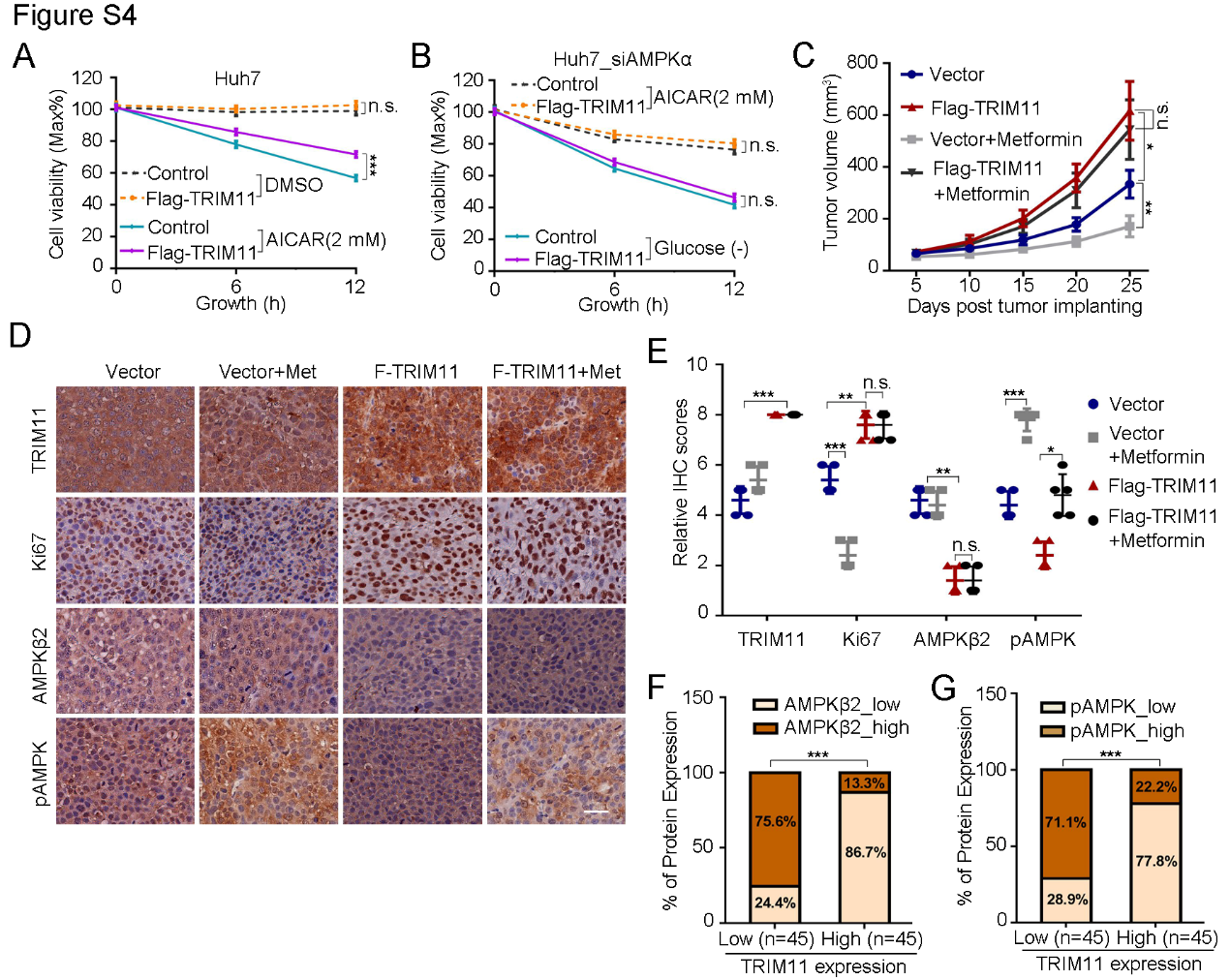
 Supplementary Figure S4. TRIM11 facilitates cell survival and tumor growth during glucose deprivation by regulating AMPK activity both *in vitro* and *in vivo*. (A) Cell viability of Huh7 cells stably expressing control or F-TRIM11, treated with DMSO or AICAR (2 mM) for 6h or 12h. (B) Cell viability of Huh7 cells stably expressing control or F-TRIM11 simultaneously transfected with AMPK siRNA, treated with AICAR (2 mM) or glucose starvation for 6h or 12h. (C) Huh7 cells stably expressing vector or F-TRIM11 were subcutaneously injected in nude mice, which fed with control (tap water) or metformin water (Met, 500mg/L) respectively. Shown are average tumor volumes over time (n = 5). (D, E) Representative images (D) of IHC staining and the relative IHC scores (n=5) (E) of TRIM11, Ki67, AMPKβ2 and pAMPK in HCC tissues of mice inoculated with vector or F-TRIM11, treated with control and metformin respectively. Scale bar, 50 μm. (F, G) Statistical data of TRIM11, AMPKβ2 and pAMPK expression in HCC tissues (TRIM11 low and high expression group, n=45). Scale bar, 50 μm. For A-C and E-G, data represent the mean ± SEM (n= 3 unless otherwise indicated). Statistical significance was assessed using two-tailed Student’s t-tests. *P< 0.05, **, *P* < 0.01; ***, *P* <0.001; n.s. not significant.


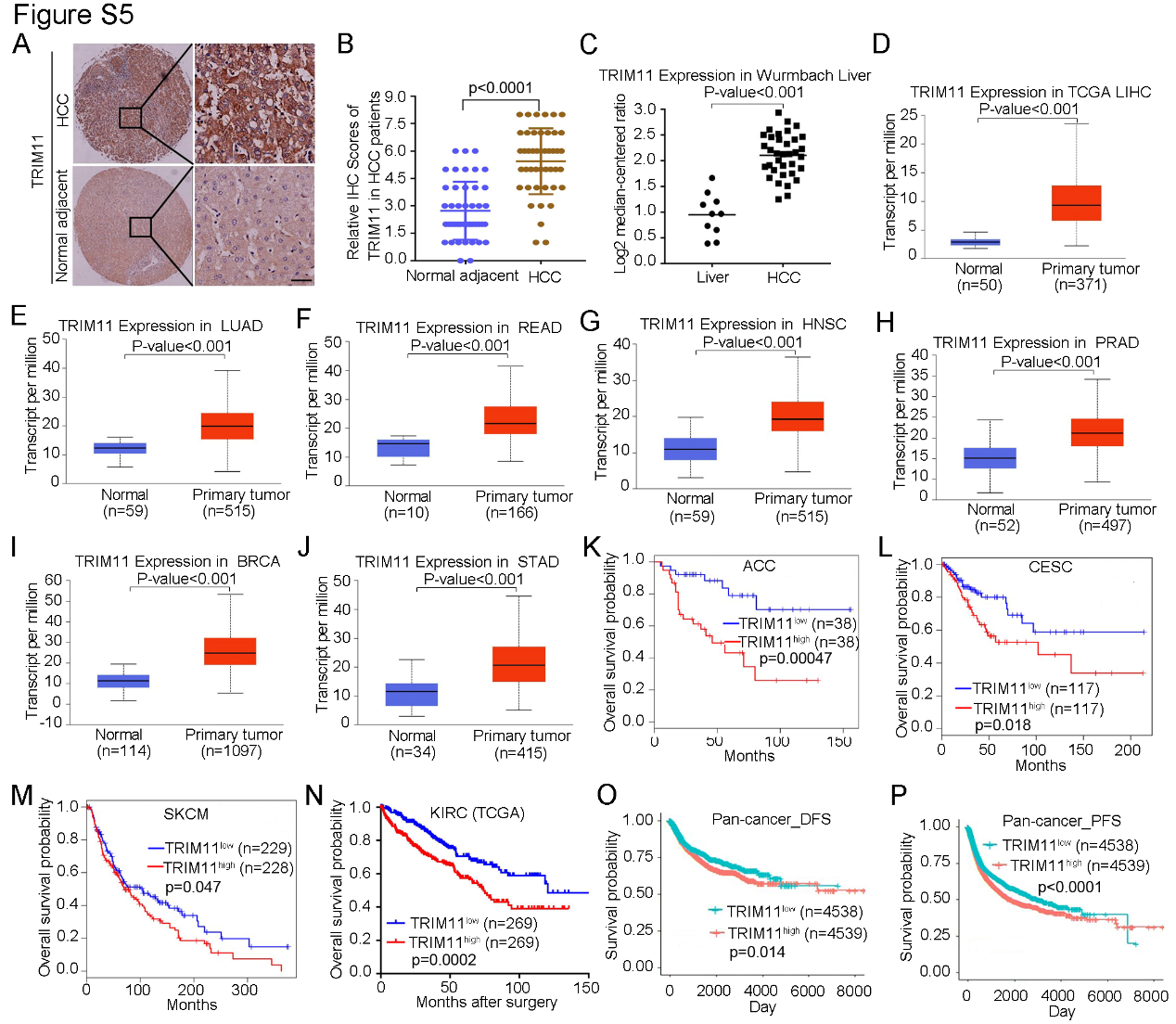


Supplementary Figure S5. Up-regulation of TRIM11 in HCC and Pan-cancer linked to poor prognosis. (A, B) Representative images (A) of IHC staining and the relative IHC scores (B) of TRIM11 in HCC tissues and adjacent normal tissues. Data represent the mean ± SEM. Statistical significance was assessed using two-tailed Student’s t-tests. (C) Comparison of the TRIM11 mRNA level between HCC and normal tissues in oncomine database. (D-J) Comparison of the TRIM11 mRNA levels analyzed from the TCGA database in human different cancers (LIHC, Liver hepatocellular carcinoma; LUAD, Lung adenocarcinoma; READ, Rectum adenocarcinoma; HNSC, Head and Neck squamous cell carcinoma; PRAD, Prostate adenocarcinoma; BRCA, Breast invasive carcinoma; STAD, Stomach adenocarcinoma;) and its corresponding normal tissues. (K-N) The overall survival and disease-free survival probability were compared between TRIM11 high and low expression in different cancer patients (ACC, Adrenocortical carcinoma; CESC, Cervival squamous cell carcinoma and endocervical adenocarcinoma; SKCM, Skin Cutaneous Melanoma; KIRC, Kidney renal clear cell carcinoma) from TCGA database. The statistical significance was assessed using two-sided log-rank test, log-rank *p* values were shown. (O, P) The disease-free (O) and progression-free (P) survival time (DFS/PFS) were compared between TRIM11 High (n=4539) and Low expression (n=4538) in the thirty-three of the most prevalent forms of cancer patients from TCGA pan-cancer cohort. For D-J, Statistical significance was assessed using two-tailed Student’s t-test; for K-P, the statistical significance was assessed using two-sided log-rank test, log-rank *p* values were shown.

**Materials and Methods**

**Vector construction and reagents.**

pEGFP-LC3 (human) was a gift from Toren Finkel (Addgene plasmid # 24920).[^1^](#_ENREF_1) mCherry-hLC3B-pcDNA3.1 was a gift from David Rubinsztein (Addgene plasmid # 40827). [^2^](#_ENREF_2) Flag-TRIM11, GFP-TRIM11, GST-TRIM11 and lentiviral vectors expressing Flag-TRIM11 or TRIM11 shRNA were previously described.[^3^](#_ENREF_3)^,^ [^4^](#_ENREF_4) TRIM11, TRIM11 mutants, AMPKβ2 and AMPKβ2 mutants were constructed into pcDNA3.1 vector with a Flag tag at N-terminal.

The following reagents were commercial obtained: Cycloheximide (CHX) (Calbiochem); AICAR, compound C, chloroquine (CQ), and Z-Leu-Leu-Leu-al (MG132) were from Sigma. Commercial antibodies include: TRIM11 (1:500, ABC926, EMD Millipore), HSP90 (1:1000, sc-69703), β-Actin (1:1000, sc-47778) and HA (1:1000, sc-7392) from Santa Cruz Biotechnology. AMPKα (1:1000, #5831), AMPKβ1 (1:1000, #4178), AMPKβ2 (1:1000, #4148), AMPKγ1 (1:1000, #4187), AMPKγ2 (1:1000, #2536), AMPKγ3 (1:1000, #2550), ACC1 (1:1000, #3676), phospho-AMPKα (1:1000, #2535), phospho-ACC1 (1:1000, #11818), mTOR (1:1000, #2972), phospho-mTOR(Ser2448) (1:1000, #5536), phospho-TFEB(Ser211) (1:1000, #37681) and phospho-ULK1(Ser555) (1:1000, #5869) from Cell Signaling Technology. TFEB (1:2000, 13372-1-AP) and ULK1 (1:2000, 20986-1-AP) from Proteintech. GFP (1:2000, #598) and LC3B (1:2000, # PD014) from MBL. Flag (1:1000, F1804) and p62 (1:1000, P0067) from Sigma. GAPDH (1:2000, NB300-221, Novus Biologicals) and HA (1:1000, 71-5500, Thermo Fisher Scientific). Secondary antibodies were conjugated to HRP (Santa Cruz) or AlexaFluor 488/568 (Invitrogen).

**Cell culture and stable cell lines generation.**

Huh7, HepG2, U2OS, HEK293T and MEF cells were cultured in DMEM (Life Technologies) supplemented with 10% FBS (HyClone) and cultured cells at 37°C with 5% CO_2_.

Firstly, lentivirus was produced by the following steps: HEK293T cells transfected with the lentiviral vectors and helper plasmids DR8.91 and VSVG. 2 and 3 days after transfection, virus-containing media were collected and concentrated by centrifuged at 10,000 g for 18 h. Then, cells were transduced using lentivirus with 8 μg/ml polybrene and selected with appropriate antibiotic to generate the appropriate stable cell lines.

**Transfection and RNA interference.**

For transfection, cells were grown to 60% confluence and then transfected with Lipofectamine® 2000 (Thermo Fisher). For AMPKα knockdown, Huh7 or U2OS cells were transfected with a siRNA pool against AMPKα (Santa Cruz) and Lipofectamine® RNAiMAX (Thermo Fisher). After transfection for 5-12 h, the medium was replaced to complete or glucose deprivation medium.

**Immunofluorescence.**

Cells were fixed with 4% PFA for 30 min and permeabilized with 0.15% Triton X-100 for 15 min. Then, cells were washed 2 times with PBS, blocked with 3% BSA for 30 min at RT followed by incubated with the indicated primary and secondary antibody for 1.5 h at RT, respectively. After washed 5 times with PBS, the Samples were mounted by medium containing DAPI (Vector Labs) and immediately observed under a fluorescence microscopy.

**Immunoblot and immunoprecipitation.**

For immunoblot, NP-40 lysis buffer (50 mM Tris-HCl, pH 8.8, 100 mM NaCl, 5 mM MgCl_2_, 1 mM NaF, 0.5% NP-40, 2 mM DTT, 1 mM PMSF, and 1x complete protease inhibitor cocktail) were used to lyse cells for 30 min on ice and then centrifuged at 15,000 g at 4 °C for 15 min to collect the supernatant. The protein concentrations were measured by Bradford assay (Bio-Rad Labs) and mixed with 6X loading buffer before boiled. For the protein half-life analysis, cells were treated with CHX (50 μg/ml) at the indicated times as the main text. These samples were resolved with 8%-10% SDS-PAGE and analyzed by western blot.

For immunoprecipitation, cells were lysed in the same above NP-40 lysis buffer and the lysates were incubated with Flag-M2 beads (Sigma) or protein A/G agarose (Thermo Fisher Scientific) for 6h at 4 °C. For AMPKβ2 immunoprecipitation, cells were lysed in SDS-containing buffer, boiled, diluted 20-fold in the NP-40 lysis buffer and incubated with protein A/G agarose for 6h at 4 °C. Then, after extensive washing, the beads were boiled in loading buffer and the samples were analyzed by western blot.

**GST pull-down assay.**

GST and GST-TRIM11 were expressed in *E. coli* BL21 (DE3) and the bacteria were cultured at 37 °C and protein expression was induced with 0.2 mM IPTG. GST and GST-TRIM11 were purified with glutathione beads, and incubated with Huh7 cell lysates at 4°C overnight. Then, after extensive washing, the beads were boiled in loading buffer and the samples were analyzed by Western blot.

**Cell viability assay.**

Cells (~2, 500 cells/well) were seeded in 96-well plates in triplicates and cultured in complete or glucose deprivation medium, treated with DMSO or drugs as indicated in the text. Cell viability were determined by measuring the OD at an absorbance wavelength of 490 nm of 3-(4,5-dimethylthiazol-2-yl)-2,5-diphenyltetrazolium bromide (MTT) (Promega) at the indicated time points.

**Glucose and Lactate Detection**

Huh7 cells infected with shRNA targeting TRIM11 were seeded in the six-well plates. After 24h, the above cells culture medium was collected. The levels of Glucose consumption and lactate production were determined using the glucose and lactate assay kit (Sigma) respectively according the manufacturer’s instructions.

**Clinical samples and immunohistochemistry**

Tissue microarray of primary HCC samples were obtained from Shanghai Tufei Biotech and US Biomax Inc. (Rockville, MD, USA). Immunohistochemical (IHC) staining was performed as the following described.[^5^](#_ENREF_5) Primary antibodies included: anti-TRIM11 polyclonal antibody (1:200; Proteintech), anti-AMPKβ2 polyclonal antibody (1:200; Proteintech) and anti-pAMPK polyclonal antibody (1:200; CST). Signals were detected using Envision-plus detection system (Dako, Carpinteria, CA, USA) and visualized following incubation with 3,3’-diaminobenzidine.

**Tumor xenograft mouse models**

Tumor xenograft mouse models were established as the following described.^6^ The tumor growth of Huh7-shNC and Huh7-shTRIM11-1&2 cells were determined following subcutaneous injection of cells into nude mice respectively (3.0×10^6^ cell/mouse, six mice/group). Four weeks post injection, the mice were sacrificed under anesthesia, and the tumor samples were then collected for further analysis. Huh7-Control and Huh7-TRIM11 cells (2.0x10^6^ cells/mouse, five mice/group) were subcutaneously implanted into the nude mice, respectively. Mice were randomly assigned to receive with or without the metformin dissolved in the drinking water (500mg/L) daily until the end of experiments. Tumor sizes were measured every five days. After twenty-five days, the tumor samples were then collected for further analysis.

All animal experiments were undertaken in accordance with relevant guidelines and regulations and were approved by the Institutional Animal Care and Use Committee at STAT.

**Data analysis.**

Data analysis was used with GraphPad Prism 7 software (GraphPad Software, USA) through the unpaired two-tailed Student’s t-test. The bands of western blot and fluorescence dots of LC3 were quantified using ImageJ (National Institutes of Health).

**References**

1. Lee IH, Cao L, Mostoslavsky R, Lombard DB, Liu J, Bruns NE, Tsokos M, et al. A role for the NAD-dependent deacetylase Sirt1 in the regulation of autophagy. Proc Natl Acad Sci U S A 2008;105:3374-3379.

2. Jahreiss L, Menzies FM, Rubinsztein DC. The itinerary of autophagosomes: from peripheral formation to kiss-and-run fusion with lysosomes. Traffic 2008;9:574-587.

3. Chen L, Brewer MD, Guo L, Wang R, Jiang P, Yang X. Enhanced Degradation of Misfolded Proteins Promotes Tumorigenesis. Cell Rep 2017;18:3143-3154.

4. Chen L, Zhu G, Johns EM, Yang X. TRIM11 activates the proteasome and promotes overall protein degradation by regulating USP14. Nat Commun 2018;9:1223.

5. Liu Y, Zhang JB, Qin Y, Wang W, Wei L, Teng Y, Guo L, et al. PROX1 promotes hepatocellular carcinoma metastasis by way of up-regulating hypoxia-inducible factor 1alpha expression and protein stability. Hepatology 2013;58:692-705.

6. Liu Y, Ye X, Zhang JB, Ouyang H, Shen Z, Wu Y, Wang W, et al. PROX1 promotes hepatocellular carcinoma proliferation and sorafenib resistance by enhancing beta-catenin expression and nuclear translocation. Oncogene 2015;34:5524-5535.
